# Attitudes of the Equestrian Public towards Equine End-of-Life Decisions

**DOI:** 10.3390/ani11061776

**Published:** 2021-06-14

**Authors:** Catherine Bell, Suzanne Rogers

**Affiliations:** Equine Behaviour and Training Association, Godalming GU8 6AX, UK; info@ebta.co.uk

**Keywords:** equine euthanasia, behaviour, welfare, stress, pain, fear

## Abstract

**Simple Summary:**

Delayed death has been identified as a key welfare concern for the U.K. horse population, leading to prolonged suffering. Previous studies have identified common reasons for delaying euthanasia, including financial cost, emotional attachment, peer pressure, negative attitudes towards killing and poor recognition of behavioural indicators of equine pain and stress. The Five Freedoms is a welfare framework that can be used to assess quality of life. We used this framework to create a survey, compiling a list of hypothetical—yet common—scenarios that would have an impact on the overall quality of life of a horse. Participants were asked to indicate to what extent each scenario would have a bearing on an overall decision whether or not to euthanise a horse, or whether it would have had no bearing at all. Participants were also asked if they had had a horse euthanised and to give the reason for such a decision. We received responses from 160 participants and found that the predominant attitude was that most scenarios had no bearing on a decision to euthanise. Principal Component Analysis collected the scenarios into a series of factors that could be labeled according to their themes, the most prominent of which were “Ethology-informed Management”, “Traditional Management”, “Emotional issues” and “Physical Issues”. Participants were most likely to consider euthanasia for physical issues and this was supported by the experiences of participants who had had their horses euthanised. Only a small number of responses also included consideration of affective and/or ethological factors, suggesting that welfare issues concerning affective state and/or behaviour are at risk of being omitted from an end-of-life decision.

**Abstract:**

A key welfare concern for the equine population in the U.K. has been identified as delayed death, leading to prolonged suffering of horses. Reasons why some horse owners fail to have their horses euthanised include financial cost, emotional attachment, peer pressure, negative attitudes towards killing and poor recognition of behavioural indicators of equine pain and stress. The Five Freedoms framework of welfare was used to build a Likert-style survey to investigate the factors underlying attitudes of horse owners towards welfare measures in an end-of-life decision. Participants were asked to respond to hypothetical welfare scenarios and to give details of any horses they had had euthanised. The survey was conducted predominantly via equestrian Facebook groups and obtained 160 participant responses. Reliability of the scale was acceptable, with Cronbach’s α=0.89. Principal Component Analysis was used to load the hypothetical scenarios onto seven factors containing 62.2% of the variance. The first four factors could be categorized according to “Ethology-informed Management”, “Traditional Horse Management”, “Emotional Issues” and “Physical Issues”. Participants were more likely to consider euthanasia for physical issues, compared with issues relating to affective state and/or ethology, although it was not clear whether this was due to disregard for welfare issues relating to mental health or failure to recognise them as such. A large number of responses stated that the scenario had no bearing on whether a horse should be euthanised, again suggesting a lack of recognition of welfare issues and their implications. When asked to state their reasons for euthanising their horses, participants cited almost exclusively physical reasons, with the exception of those citing dangerous behaviour. Only a small number of responses also included consideration of affective and/or ethological factors, suggesting that welfare issues concerning affective state and/or behaviour are at risk of omission from end-of-life decisions.

## 1. Introduction

In 2016, Horseman et al. identified four priority welfare concerns for the equine population in the U.K. [1], one of which was delayed death, leading to potential suffering and protraction of existing suffering of the horses affected. It was hypothesized that owners were reluctant to euthanise due to reasons such as financial cost, emotional attachment, peer pressure and negative attitudes towards killing. Another welfare concern highlighted was the poor recognition of pain and stress by caregivers; the inability of many horse owners to recognise behavioural indicators of fear and stress was explored further, using video footage featuring a variety of horse training styles [2]. It follows that poor understanding and recognition of some welfare factors could be an additional causal factor in prolonging end-of-life suffering.

Animal welfare is commonly assessed in terms of the “Five Freedoms” framework, developed in particular for application to farm animals (FAWC, 1993; cited in [3]) but has also been applied to horses. The list is non-hierarchical and all “freedoms” are considered equally important. Under this framework, good welfare requires:

- Freedom from hunger and thirst
- Freedom from discomfort
- Freedom from pain, injury or disease
- Freedom to express (most) normal behaviour
- Freedom from fear and distress

The alternative, “Five Domains” model [4,5] has also been widely adopted in order to allow a degree of “welfare compromise”. With a graded consideration of welfare that allows for positive and negative experiences in a day, rather than the complete absence of poor practice, the Five Domains can be considered more practical and gives rise to an assessment of the impact of human activities on animal affect. For example, it has been used to assess Thoroughbred welfare and develop the Thoroughbred Welfare Assessment Guidelines of New Zealand Thoroughbred Racing [6]. It has also formed the basis of the Equid Assessment, Research and Scoping welfare assessment tool, an attempt to standardise various other assessment tools into a single instrument that could be suitable for all equids in different settings [7]. The Five Domains model is often considered to set a higher standard than the Five Freedoms, since it requires animals to thrive and have positive experiences, rather than merely survive. Further comparison of Freedoms vs. Domains is also available [8].

Use of the Five Freedoms framework was retained for the work presented here because euthanasia requires a definite decision due to reduced welfare, rather than compromising on acceptable ongoing welfare. The Five Freedoms framework has been used to evaluate tools used for assessment of equine welfare, in terms of both their ease of use and their limitations [3]. For example, hunger can be assessed via visual body condition scoring and/or availability of food, and discomfort can be assessed by availability of other resources such as shelter. There is an extremely wide variety of tests to diagnose pain and discomfort, for example pain/lameness scales, gait analysis and physiological measures, such as respiration and capillary refill time [3]. The ability of the horse to express normal behaviour can be ascertained through observation of the horse in the context of time budgets, rebound behaviour, demeanour and attitude, and whether the horse behaves in a manner indicative of reduced affective state. Fear and distress were cited as being measurable via physiological markers such as cortisol and heart rate variability [3]. More recently further means of assessing pain and stress/fear have become available, for example the pain ethogram [9] and eye blink rate [10] respectively.

While the variety of tools cited is available to veterinarians and experts in welfare, the majority of these tests are unlikely to be applied by horse owners, perhaps due to lack of awareness or lack of veterinary expertise. For example, lameness analysis and heart-rate variability require expert input [3], there is evidence to suggest that owners do not always recognise disease or health issues [11] and management practices such as long-term stabling—and the horses’ abnormal behavioural responses to such practices—have become normalized in the equine industry and owners commonly fail to recognise the early signs of pain, fear and stress [1,2]. Therefore, although there is useful reassurance that the Five Freedoms framework is a suitable means of assessing equine welfare [3], it is not sufficient to assume that horse owners will necessarily apply it. Owner attitudes towards these welfare measures also need to be accounted for so that effective outreach and engagement approaches can be used to encourage horse owners to better understand their horses’ welfare needs.

Studies of owner attitudes towards welfare have been conducted previously, although not specifically in the context of the Five Freedoms. For example, owners were surveyed regarding a single specific management practice relevant to maintaining freedom from fear and distress and the freedom to express normal behaviours: keeping horses in groups [12]. In this study, a web-based survey was used to ask owners about their horse-keeping practices and their attitudes towards such practices. Whilst 86% strongly agreed that keeping horses in groups was good for welfare and 92% recognised that horses need equine company, this was not matched by practice. Only 47% of the horses cared for by the respondents were kept in groups for 24 hours per day compared with 45% stabled alone for either day or night. Horses spending the least time in groups tended to be stallions and those used for competition; reasons cited for ongoing separation included the risk of injury to horses and difficulties feeding group-kept horses. Rebound behaviours (e.g., high speed movement following confinement) were cited [3] as an indicator that horses were not free to express their normal behaviours and we note that it is these rebound behaviours and poor socialization that commonly cause the equine injuries feared by owners. It was concluded that there is a great need for education to mitigate owner concerns about implementation of higher welfare management, so that owners can align their behaviours regarding the way they manage their horses with their attitudes regarding equine welfare [12].

Owner attitude towards stereotypies has also been studied, due to the potential welfare implications inherent in such behaviour [13]. Crib-biting has a wide range of possible aetiology, including lack of opportunity to forage, gastrointestinal issues, and isolation from conspecifics, yet nearly half of survey respondents believed it to be caused by horses copying one another. Not only is there currently little evidence to suggest that horses learn to crib-bite from other horses, the survey also established that only 1% of horses mentioned in the survey started to crib-bite after the arrival of a crib-biting horse, counter to the assumptions of some of the respondents.

Other studies have retrospectively researched owner attitudes towards euthanasia. For example, a survey of owners of geriatric horses sought to better understand reasons underpinning a decision to euthanise and the risk factors that increase the likelihood that such a decision will be made [14]. Of 118 cases of mortality, 94% were euthanised, mostly for reasons of lameness or colic. For acute cases it was noted that veterinarian advice was a key factor in the decision to euthanise, whereas in more chronic cases the owner’s perception of quality of life became more important. While “quality of life” was not defined, risk factors included perceived pain, weight-loss and an increasing limitation of daily activities, such as getting up after lying down, difficulty eating and reduction in time spent lying down. In an alternative approach, respondents were assessed according to personality type and asked about their reasons for opting for euthanasia [15]. Factors relating to horse health were most commonly cited, predominantly pain-related conditions, although depression was also cited as an important factor by a minority of 2.7%. Anticipated quality of life, in the context of ongoing management, was also cited highly but without specificity as to what factors constituted sufficiently low quality of life that euthanasia was required. Veterinarian opinion was also cited as a key reason to euthanise, with potential implications for attitudes towards whose responsibility the decision should be. The horse–owner relationship during key events, including euthanasia, has also been explored, noting that the decision to euthanise is made particularly difficult, sometimes to the point of impacting welfare, by the relationship and strong attachment owners have with their horses [16]. In keeping with other studies, quality of life, health and prognosis were found to be relevant to the decision to euthanise; temperament of the horse was also cited as influencing timing of euthanasia.

In summary, the Five Freedoms is a well-established welfare framework that forms a useful instrument upon which to base a survey regarding attitudes towards equine end-of-life decisions. There is existing research into the equestrian public’s attitudes towards welfare and reasons underpinning their decisions to euthanise, but other studies discussed above have already indicated the potential for discrepancy between attitudes towards welfare and actual practices implemented and it is very easy for owners to create a ’post hoc’ justification for their decision. Thus it is useful to establish an understanding of welfare factors that people consider important in advance of actually making the decision to euthanise. Research conducted to date would suggest that physical welfare factors such as a broken leg or acute colic would be considered appropriate grounds for euthanasia on account of the extreme suffering involved [14]; however, it is the more subtle welfare factors that are likely to lead to the *delay* of euthanasia and subsequent suffering. Here we consider these more subtle factors, including mental health and the extent to which ethological needs are met, in addition to the physical factors.

## 2. Materials and Methods

### 2.1. Materials

A survey incorporating 30 welfare scenario statements was created in order to probe participant attitudes towards welfare surrounding end-of-life decisions. The 30 scenarios were developed by the authors, using the Five Freedoms as a guide, with six scenarios per freedom; they were chosen to be independent of one another and reflect a range of good or compromised welfare situations that domestic horses commonly experience, both ’in the moment’ and over extended periods of time (Table 1). Some scenarios were clear as to whether or not they were good welfare, for example, lameness fairly unambiguously reduces welfare [15]. Other scenarios were more debatable and would be more likely to probe a range of attitudes, for example whether being kept alone, albeit with other horses in view, is acceptable welfare [12]. For each freedom the scenarios were loosely split between good and compromised welfare but not so much so that there was an obvious “correct answer”. For example, in order to balance the good and compromised welfare scenarios for “Freedom from pain, injury and disease” we could have used a scenario “The horse moves freely and with an even gait”, or “The horse has never experienced colic” but we anticipated that these would have too obviously been recognised as good welfare. Instead we considered that the wider range of compromised welfare scenarios for this freedom was more useful to probe a variety of participant attitudes.

The survey was prepared and published via Qualtrics (2020). It asked two initial demographic questions:aAge and gender of participant. The main section of the survey then began with some explanatory text and an example welfare scenario, “The horse was turned out with a companion who frequently bullied him”. It was explained that such a scenario would be unlikely to trigger a euthanasia decision in isolation, but that we were more interested in whether it would contribute to an overall quality of life assessment and, consequently, make the participant more or less likely to opt for euthanasia. It was also explained that, while there are alternative solutions — for example, in this case finding alternative companions — they should not be considered an option here. While we recognised that the restriction on making improvements would be considered improbable by the more welfare-minded respondents, it was required because the scenarios reflect situations routinely experienced by horses (e.g., [17,18]).

The scenarios for participants to consider were then listed in random order: Participants were asked to state on a Likert-type scale whether they considered each scenario to strongly increase, slightly increase, slightly decrease or strongly decrease the likelihood that they would decide to euthanise the horse in each case. A neutral “would have no bearing on the decision” option was also included. A final free-text question was added, asking whether the participant had previously made a decision to euthanise a horse and, if so, for what reason.

The prepared survey was sent to five horse owners, chosen to reflect a range of welfare knowledge, in order to test for clarity; they responded to say that they found the survey to be self-explanatory.

### 2.2. Participants

Participants were recruited by sharing the online survey link in equestrian Facebook groups. It was requested that they were over 18 years old and either owned or loaned a horse, currently or in the past. In an attempt to obtain a large but random sample that was representative of the equestrian community, Facebook groups were selected on the basis of being large (>3000 members) and generic in terms of equestrian interest; for example equine welfare groups were not included in order to mitigate bias towards welfare-oriented horse owners. Four suitably large and generic groups were selected: Horse and Hound, Chitchat and Tack, Surrey Horse and Pony, Hampshire Horse Riders. While instead this may have risked biasing against more welfare-oriented horse owners, we note that the survey was shared further on Facebook by friends and welfare-oriented colleagues, somewhat redressing the balance. A small number of additional horse-owning participants were obtained via The Open University DE300 Surveys Facebook group. The survey was available for 30 days during March 2020.

Ethical considerations followed the Code of Ethics and Conduct of the British Psychological Society [19], with ethical approval obtained via the DE300 ethical approval processs at the The Open University. Participants were given an introductory briefing stating the purpose and content of the study. They were informed of their rights to anonymity and data protection, including data storage and disposal following the end of the study, as well as their freedom to withdraw at any point. Participants were subsequently asked for their informed consent via a ’forced choice’ response. All other questions were optional. Given the sensitive nature of the subject, it was made clear in the introductory material that the topic was potentially upsetting. Likewise the final question was also phrased carefully and included a clear “prefer not to answer” option. Finally, a debriefing was provided following completion of the survey.

### 2.3. Procedure

The results were exported to SPSS (IBM SPSS Statistics for Mac version 25, 2017) for analysis. The inclusion of positive and negative welfare statements and choice of Likert options removed the need for reverse scoring of responses, whilst still reducing the likelihood of acquiescence response bias. The data were checked for duplicates and assessed for completeness of responses. Reliability was checked by calculation of Cronbach’s α, whereby survey items were cross-correlated with one another and inter-item correlations required to be *r* > 0.3 [20]. A value of α > 0.7 was considered to indicate a high level of internal consistency. Finally, an exploratory factor analysis, Principal Component Analysis (PCA) was used to extract factors underlying participants’ attitudes towards the welfare scenarios. The sample size was checked using the Kaiser–Meyer–Olkin test of sampling adequacy and the number of correlations was checked to be sufficient using Bartlett’s test of sphericity. The number of factors retained was kept as low as possible, using both scree plot analysis and retention of eigenvalues greater than 1. By categorising according to the Five Freedoms, we wrote the scenarios to be independent of one another and therefore used varimax rotation to load the items onto each factor, maximising the loading of each variable onto a factor, whilst minimising its loading onto others [20,21].

Figures were plotted using Microsoft Excel. Responses to the single, qualitative question about reasons for euthaniasia were clustered manually by generalised theme, such as lameness, dental issues or dangerous behaviour.

## 3. Results

The survey received 160 responses, of which none were duplicates and a total of 11 answers to individual questions were missing. Of the 160 participants, 159 provided their ages, ranging from 18 to 75, with a mean age of 42.2 years and standard deviation 14.0. A breakdown of age-ranges shows a reasonably even split across ages from 18 to 55 years but then decreases for participants in their late 50 s and above (Table 2). There was a strong female bias to the responses, with 152 participants identifying as female (95.0%), 7 as male (4.4%) and 1 as “other” (0.6%).

### 3.1. Response to Scenarios

The data were inspected in order to obtain a general understanding of how participants responded to the welfare scenarios presented. First, it was noted that the neutral answer “Has no bearing on my decision” was selected by over 50% of participants for 22 of the 30 scenarios. Secondly, with the exception of the “no bearing” responses, nine scenarios were considered unambiguously to reduce the chances of deciding to euthanise, for example “The horse has access to forage 24/7”. Thirdly, only one or two scenarios were unambiguously considered to increase the chances of deciding to euthanise, in particular “The horse is visibly lame at walk”. Finally, the remaining scenarios seemed to split the response as to whether euthanasia would be deemed more or less likely.

There were insufficient responses from men, and people who did not identify as male or female, to do a statistical comparison with the majority female responses. However a basic comparison showed that the mean number of times the “has no bearing” option was selected by those identifying as male was 19.0 (standard deviation 6.1) and compared with 19.1 (standard deviation 6.6). Therefore the data were treated as a single sample for the duration of the study.

An initial reliability analysis on all 30 survey scenarios gave an acceptable value for Cronbach’s α of 0.89 (Table 3). Inspection of the item statistics suggested that most items are worthy of retention. Two scenarios were possible exceptions to this: “The horse is visibly lame at walk” and “The horse is closely bonded with a companion” gave respectively lower and higher mean scores than the other items. Inter-item correlations showed that both items still correlated significantly (r>0.3) with some—but not all—other items. Corrected item-total correlations were not significant, with r<0.2 for both, but their individual exclusion did not increase the value of α above 0.89. Two other items, “The horse has competed all his life but can no longer do so” and “The horse is able to lie down and get up again” also had non-significant corrected item-total correlations but again did not have an impact on the overall value of α. A re-analysis excluding both “The horse is visibly lame at walk” and “The horse is closely bonded with a companion” (but retaining “The horse has competed all his life but can no longer do so” and “The horse is able to lie down and get up again”) resulted in an overall α=0.90, suggesting that trial removal of these two items in the subsequent PCA would be worthwhile.

PCA with varimax rotation was conducted initially on the complete set of 30 scenarios. Inspection of the correlation matrix showed a range of correlation coefficients 0.3 < r < 0.9, suggesting that PCA is an appropriate appropriate. The Kaiser–Meyer–Olkin measure was 0.85, above the acceptable threshold of 0.6 and indicating adequate sample size for factor analysis. Bartlett’s test of sphericity was significant ($\chi^{2}$ (435) = 1996.815; *p* < 0.001) confirming that there are enough correlations for factor analysis. Both scree plot analysis and consideration of eigenvalues greater than unity suggested that there were seven factors that should be retained, explaining 62.2% of the variance. Forcing retention of just the first four key factors caused the variance to drop to just 50% whereas increasing the variance to 68% required nine factors. Therefore seven factors seemed a suitable intermediate value, retaining all eigenvalues greater than unity.

**Figure 1:**
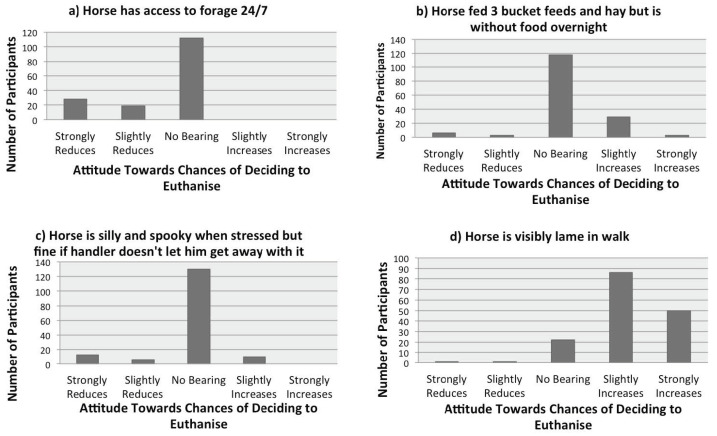
Sample items from the key four factors, labelled (as described in Section 4) (**a**) Ethology-informed Management, (**b**) Traditional Horse Management, (**c**) Emotional Issues, (**d**) Physical Issues. The first three clearly show that the majority of participants do not consider that these scenarios have any bearing on a decision to euthanise.

Repeating the analysis with omission of the two scenarios highlighted by the reliability analysis made a small (1%) increase in the variance contained in the first seven eigenvalues. Otherwise there appeared to be little difference. All items were therefore retained for subsequent consideration so as to benefit from welfare information relating to those scenarios. The seven factors identified by the PCA are shown in Table 4 and examples of each of the first four factors are shown in Figure 1.

### 3.2. Reasons for Euthanasia

The free-text qualitative question, about whether they had had a horse euthanised and for what reason, was answered by 123 of the respondents, in total providing usable data for 211 horses. Responses by three participants could not be used as it was not made clear how many horses were being discussed or did not directly answer the question. Some participants (19) justified their decision on the basis of advice from a professional, typically a veterinarian, recommending euthanasia; conversely, one respondent regretted not euthanising sooner but had instead followed veterinary advice to attempt to prolong life. Others were much more able to “own” the decision with comments such as “I won’t have a horse in pain”. The majority just stated the relevant ailments.

**Figure 2:**
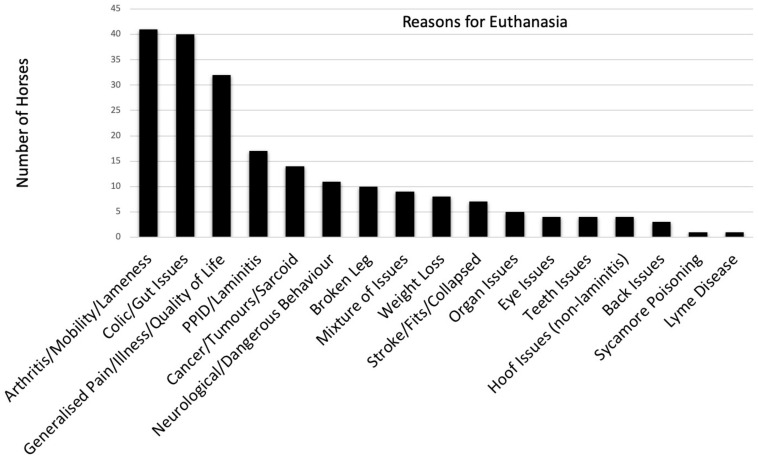
Responses to the free-text question regarding reasons for euthansia. The majority of the 211 horses discussed were euthanised for predominently physical reasons linked to “Freedom from pain, illness and disease”, with the exception of the 11 horses euthanised for dangerous behaviour (which may well have been linked to undiagnosed pain nonetheless [9]). A small number of respondents included additional considerations of mental health and other “freedoms” in their decision, as discussed in the text.

In almost all cases, the reasons given for euthanasia were predominantly for physical issues that would have otherwise contravened “Freedom from pain, injury and disease” (Figure 2). Most gave very specific answers, with colic (19%) and various types of arthritis and lameness (19%) being the most common primary reasons for euthanasia. 15% gave less specific answers with “pain”, “old age” and/or “quality of life” cited as reasons. The term “quality of life” was not defined but used in a colloquial, generalised sense. Other horses (4%) had a multitude of physical issues and it was not clear whether there had been a particular “final straw” or dominant condition that triggered the decision to euthanise.

Eleven horses were euthanised due to behaviour described as “dangerous” or “aggressive”, sometimes citing a brain tumour or neurological issue but it was not clear whether this was a formal diagnosis or a presumption based on failure to find an alternative behavioural aetiology. Two responses included the word “depression” and a third used the expression “the sparkle left” in the context of horses with Pituitary Pars Intermedia Dysfunction (PPID), soundness issues and “health problems”; a further three horses were described as having physical issues that “impacted mental health” or “caused distress”. Five responses referred to horses being unable to cope with (additional) box rest and two mentioned loss of a companion; one respondent described two mobility-impaired veteran horses who were euthanised together due to being bonded as a pair and a second respondent referred to a horse suffering (amongst various physical issues) weight loss and stress following the loss of a companion.

Finally, two respondents used the free-text answer to comment on the perceived lack of relevance of the scenarios to an end-of-life decision: “Many of the scenarios you describe have no or little bearing on putting a horse to sleep, they are simply different situations that can be remedied with correct management and/or veterinary care and training” and “None of your questions were relevant to why euthanasia should occur. Sorry”. Similar remarks were added to the Facebook posts by people who had opted to abort the survey. Such comments add to the many “no bearing” Likert responses and are discussed in the next section.

## 4. Discussion

PCA is an exploratory factor analysis that reduces the number of dimensions of a dataset, whilst preserving its statistical information, thereby identifiying themes within the dataset that can be followed up in more targetted research [21]. The most common response to the survey was that most scenarios did not have any bearing on a decision to euthanise (Table 4) and that, consequently, the small numbers of other response should not be overinterpreted. Nonetheless, PCA conducted on the survey data identified a series of seven factors containing 62.2% of the variance. The first four factors, onto which were loaded 22 of the 30 scenarios, could be clearly defined in terms of aspects of equine welfare. We have labelled them according to the predominant nature of the scenarios contained: (1) Ethology-informed Management (albeit with two exceptions), (2) Traditional Horse Management, (3) Emotional Issues, (4) Physical Issues. Details of these factors are listed in Table 5, including the relevant Freedoms and a summary of the predominant survey responses for each.

“Ethology-informed Management” was the strongest factor, containing the most variance and loaded by the greatest number of items. Most of the scenario items relate to domestic horses living under conditions which approximate to those experienced by feral horses [22] and are often considered to be examples of “good welfare” ([23] and references therein). The two items not relating to ethology are more open to interpretation, seemingly at odds with the otherwise welfare-centered items, but appear to reflect the perception amongst the participants that they too are desirable, on account of this factor containing the highest numbers of “strongly/slightly reduces” responses. Firstly, compliance is typically perceived to be a sign that the horse is well-trained and living in a low-stress environment but it can also be misinterpreted, with horses bordering on a state of learnt helplessness and given no opportunities to make their own decisions [2,24]. Secondly, perception of a “clean cosy stable” as a positive state is fairly anthropomorphic, with horses more ethologically suited to a life outside with freedom of movement in a social group. While many people recognise that group-living is beneficial to equine welfare, it is common not to implement such a regime [12]. Since the current study did not compare attitudes with actual practices, it is not possible to comment on this; however, using a similar Facebook survey strategy, it was also found that even when owners recognise that a horse is in a state of fear or stress, a substantial number consider it acceptable [2]. Thus it seems likely that recognition is not the only barrier to redressing the problems associated with delays to euthanising a horse whose welfare is compromised, and appropriate action to address the problem also needs to be facilitated.

“Traditional Horse Management” included items that can be considered widespread practice but perhaps questionable in terms of welfare [18]. The items suggested a routine-based—rather than ethology-based—approach to management, with practical convenience to the human prioritised above considerations of the horse’s perspective. This factor included high numbers of participants who thought the scenarios had no bearing on a decision to euthanise, although there was a small number of exceptions to this on both sides of the increase/decrease chances of euthanasia dichotomy.

The third and fourth factors showed a clear demarcation between scenarios featuring emotional issues versus physical issues. The physical issues factor was the only one for which “has no bearing” was not the dominant survey selection and, instead, the likelihood of euthanasia being slightly/strongly increased. Mild lameness (“sometimes unlevel on hard ground”), perhaps of the sort caused by mild arthritis, was deemed more euthanasia-worthy than what could be high levels of prolonged stress (e.g. “The horse can be silly and spooky when stressed but is fine if the handler doesn’t let him get away with it”), prompting ethical discussions as to whether the participants were more concerned about mild physical issues than impaired mental health or, alternatively, did not recognise the implications of these scenarios for mental health [2].

The final three factors are somewhat mixed and may be worthy of omission or rephrasing for follow-up study. Equally, however, they reveal insight into the attitude of some horse owners. Factor 5 contains two scenarios relating to physical health, firstly treatment for back pain and secondly, the use of a collar to prevent wind sucking (a stereotypical behaviour commonly associated with gut ulcers and stress; physical prevention of the behaviour by using a collar increases the degree of stress [25]). Yet while the back treatment has the potential to be genuinely therapeutic, more participants stated that they would be more likely to euthanise a horse with treated back pain than the horse being kept in a state of likely stress and gut pain. It may be that participants thought that the horse was no longer able to be ridden and that that equated to a life not worth living, or that they thought that the pain could not be resolved. Alternatively, it may betray an ignorance of what a horse who wind sucks actually experiences [13].

The finding of previous studies that pain-related conditions, particularly lameness and colic, are the most common reasons for euthanising horses [14,15] is confirmed by this study, in both the responses to the hypothetical scenarios and the free-text descriptions of participant experiences. There were only 11 horses euthanised for whom physical ailments were not mentioned, citing instead, “dangerous behaviour”; we note that such behaviours are commonly caused by pain-related conditions [9]. While this possibly reflects an underlying recognition of compromised emotional welfare and desire to end further suffering, the fact that the behaviour escalated to a dangerous level suggests poor recognition and/or resolution of precursor behavioural indicators [2]. Intervention then may have taken place only once human safety became compromised and the horse could no longer be “used”.

However, we found that there was a small number of exceptions where participants did consider the mental health of their horses. Euthanasia due to a recognition that horses would struggle with necessary box-rest or loss of a companion indicated that “Freedom from fear and distress” and/or “Freedom to express normal behaviour” were considerations that were included in the overall decision to euthanise. The scenario “The horse spends a lot of the day standing quietly with head lowered and eyes half closed” can probe depression and, with 15.6% of participants saying it strongly increases their chances of deciding to euthanise, this would seem much higher than the previous finding of 2.7% who actually euthanised for depression [15]. However, this scenario can equally apply to a horse in severe pain [9]. Depression could also be probed via the scenario “The horse is always compliant” although, since only one participant selected this scenario as being likely to increase the chances of euthanasia, it is more likely that most people interpreted the scenario as a desirable welfare state relating to contentment. While it is unlikely that mental health issues alone will be a common reason for euthanasia, it was reassuring to see that at least a small number of participants had explicitly recognised that depression, distress, loss of companions and box rest were significant factors reducing equine quality of life.

The selection “has no bearing” on a decision to euthanise is of key interest on account of its high frequency. The vast majority of the scenarios presented to participants were indeed positive or negative welfare so one must question whether participants failed to recognise them as such, or perceived them to be sufficiently insignificant that they did not feature in an end-of-life welfare assessment. The comment suggesting that the scenarios were all issues that could be addressed is true, to some extent, because none of the scenarios would have warranted euthaniasia in isolation. However the scenarios were selected on the basis that they reflect reality for many domestic horses, and the ensuing compromised welfare is normalised [18]. When compounded together, the overall welfare can become very poor.

There are alternative ways in which we might interpret the survey results, particularly the possibility that participants misunderstood the task, in which case there may be an issue with validity that should be checked in any future follow-up study. The study was designed to minimise this, requesting early feedback from five trial participants and providing an example scenario with advice on how to answer the survey. The low number of missing data points (11 for all participants/scenarios), as well as the consistency between Likert responses and retrospective open responses, may reflect widespread understanding. Nevertheless, the desire to resolve issues, rather than increase the likelihood of euthanasia may have remained strong, despite the instructions saying to ignore, reflecting a greater desire to mitigate suffering than the many “no bearing” responses might suggest. Based on the “Physical Issues” factor, however—for which “has no bearing” did not dominate—it would seem that participants were wanting to select euthanasia for a single physical catastrophic and possibly unresolvable event, rather than a more nuanced decline in welfare towards end-of-life. While overall the scenarios comprised a fairly even ratio of input-based measures (e.g., management), to output-based measures, (e.g., physical or behavioural consequences), the “Physical Issues” factor contained only output measures; consequently the increased likelihood of euthanasia for this factor may have reflected ease of recognition of more tangible signs of suffering, compared with the more implicit nature of input-based measures. However, the fifth factor also included increased likelihood of euthanasia and all scenarios loaded were input-based measures, suggesting that the input/output nature of the scenarios cannot directly explain the results. A final consideration that may have skewed the results was that participants may have felt defensive that the survey implied some common practices were potentially welfare concerns, and to the extent that they should contribute towards a decision to euthanise—there were no comments to this effect and the survey was designed with a mixture of good and compromised welfare scenarios to counter this. However we cannot discount the possibility.

## 5. Conclusions

The equestrian public appears to consider a limited set of welfare factors when assessing quality of life, in advance of deciding to euthanise their horses. Physical issues, including even mild lameness, are more likely to factor in an end-of-life decision than issues relating to mental health, and horse owners are less likely to account for subtle welfare issues, potentially leading to the delay of euthanasia and prolonged suffering. Even if issues are noticed, it cannot be assumed that the implication of those observations will be understood or considered relevant to the welfare of the horse. The results of this study should be used to inform educational welfare programs and identify themes to be followed up in more targetted future research.

## Figures and Tables

**Table 1:** Welfare status of each of the 30 scenarios (although some are more debatable than this table suggests, for example attitudes towards rugging). Scenarios are listed according to the Five Freedoms, and presented as pairs of good or compromised welfare where possible.

Freedom	Good Welfare	Compromised Welfare
**Freedom from hunger and thirst**	The horse has access to forage 24/7.	The horse has 3 bucket feeds and hay each day, but is without food between 9 p.m. and 7 a.m.
	The horse has access to a range of browsing (is able to eat trees, shrubs, leaves etc.).	The horse chews his stable door but the behaviour is managed by painting the door with bitter-tasting paint.
	The horse can’t eat hay due to teeth issues but has free access to a range of alternative forage-based feeds.	The horse has plenty of hay but doesn’t maintain weight.
**Freedom from discomfort**	The horse always has free access to a field and soft bedding.	The horse’s field gets very muddy in winter and rutted in summer.
	The horse doesn’t have a stable but the field is sheltered by trees.	The horse always comes in to a clean, cosy stable overnight and some of the day.
	The horse is never rugged unless the weather is particularly cold and wet.	The horse is rugged consistently from October to April.
**Freedom from pain, injury and disease**	The horse has regular treatments for a sore back.	The horse is becoming increasingly aggressive but is manageable if the handler is firm.
		The horse spends a lot of the day standing quietly with head lowered and eyes half closed.
		The horse is visibly lame at walk.
		The horse sometimes looks unlevel on hard ground.
		The horse wears a collar to prevent him wind sucking.
**Freedom to express (most) normal behaviour**	The horse lives in an established herd of mixed-age horses.	The horse is turned out alone but can always see other horses.
	The horse is able to lie down and get up again.	The horse gets plenty of sleep but always standing up.
	The horse has varied forms of enrichment in the field.	
	The horse has competed all his life but can no longer do so.	
**Freedom from fear and distress**	The horse is closely bonded with a companion.	The horse has separation anxiety for a few hours each weekend when
		his companion is competing.
	If the horse seems anxious then the handler lets him have a break.	The horse can be silly and spooky when stressed but is fine if the handler doesn’t let him get away with it.
		The horse needs physical reminding to keep out of my space.
		The horse is always compliant.

**Table 2:** Number of participants in each age range (years).

	18–25	26–35	36–45	46–55	56–65	66–75
**Number**	35	31	35	38	14	6

**Table 3:** Reliability assessment showing two calculations of Cronbach’s α for N = 153. The second calculation excludes the two items “The horse is visibly lame at walk” and “The horse is closely bonded with a companion”.

	30 Items	28 Items
**Cronbach’s α**	0.89	0.90
**Scale mean**	95.0	84.8
**Scale variance**	108.7	102.0
**Standard Deviation**	10.4	10.1
**Item Mean**	3.17	3.03
**Item Range**	6.47	1.70

**Table 4:** Survey responses for each scenario and the factors onto which they were loaded by the PCA. Columns list the numbers of participants who selected “Strongly Reduces”, “Slightly Reduces”, “Has No Bearing On”, “Slightly Increases” or “Strongly Increases” the likelihood that they would decide to euthanise. Scenarios are ordered according to the factor in which they were placed by the PCA. Scenarios which had missing data (one in each case) are indicated by (1).

	Str.	Sl.	No	Sl.	Str.
Scenario	Red.	Red.	Bearing	Inc.	Inc.
**Factor 1: 16.6% of variance after rotation**					
The horse has access to forage 24/7 (1)	28	19	112	0	0
The horse has access to a range of browsing (e.g., is able to eat trees, shrubs, leaves etc.)	25	20	114	1	0
The horse always has free access to a field and soft bedding	32	29	99	0	0
The horse is never rugged unless the weather is particularly cold and wet (1)	12	8	136	3	0
The horse has varied forms of enrichment in the field (1)	32	33	94	0	0
The horse lives in an established herd of mixed-age horses	23	20	116	1	0
The horse is able to lie down and get up again	50	61	49	0	0
The horse always comes in to a clean, cosy stable overnight and some of the day (1)	16	11	128	4	0
The horse is always compliant	26	14	119	0	1
The horse is closely bonded with a companion	14	37	95	14	0
**Factor 2: 10.9% of variance after rotation**					
The horse chews his stable door but the behaviour is managed successfully by painting the door with bitter-tasting paint (1)	7	4	127	18	3
The horse is rugged consistently from October to April (1)	6	4	146	1	2
The horse has 3 bucket feeds and hay each day. However, is without food between 9 p.m. and 7 a.m. (1)	6	3	118	29	3
The horse is turned out alone but can always see other horses (1)	7	2	137	12	1
**Factor 3: 9.4% of variance after rotation**					
The horse can be silly and spooky when stressed but is fine if the handler doesn’t let him get away with it (1)	13	6	130	10	0
If the horse seems anxious then the handler lets him have a break	11	6	133	10	0
The horse needs physical reminding to keep out of my space	8	2	137	13	0
The horse has separation anxiety for a few hours each weekend when his companion is competing	6	3	119	31	1
**Factor 4: 7.9% of variance after rotation**					
The horse is becoming increasingly aggressive but is manageable if the handler is firm	2	5	47	91	15
The horse is visibly lame at walk	1	1	22	86	50
The horse has plenty of hay but doesn’t maintain weight	1	2	51	97	9
The horse sometimes looks unlevel on hard ground	5	4	72	78	1
**Factor 5: 7.3% of variance after rotation**					
The horse wears a collar to prevent him wind sucking (1)	6	2	109	35	7
The horse can’t eat hay due to teeth issues but has free access to a range of alternative forage-based feeds (1)	7	22	52	70	8
The horse has regular treatments for a sore back	4	7	74	70	5
The horse’s field gets very muddy in winter and rutted in summer	6	2	114	37	1
**Factor 6: 5.3% of variance after rotation**					
The horse spends a lot of the day standing quietly with head lowered and eyes half closed	3	3	69	60	25
The horse gets plenty of sleep but always standing up	4	4	96	50	6
The horse doesn’t have a stable but the field is sheltered by trees	16	5	127	12	0
**Factor 7: 4.7% of variance after rotation**					
The horse has competed all his life but can no longer do so	14	2	105	35	4

**Table 5:** Characteristics of the factors identified using PCA. “Label” refers to the names we gave each factor, “Freedom From/To...” lists the Freedoms relevant to the scenarios loaded on each factor and “Attitude Towards Likelihood of Euthanasia” reflects the most common Likert responses, as listed in Table 4.

Factor	Label	Freedom From/To…	Attitude Towards Likelihood of Euthanasia
1	Ethology-informed Management	Hunger + Thirst Discomfort Express normal behaviour Fear + Distress	No bearing Strongly/slightly reduces
2	Traditional Management	Hunger + Thirst Discomfort Express normal behaviour	No bearing Slightly reduces/increases
3	Emotional Issues	Fear + Distress	No bearing
4	Physical Issues	Pain, Injury + Disease Discomfort Hunger + Thirst	Strongest increase Lowest selection of ’no bearing’
5	Mixed Welfare	Pain, injury + Disease Hunger + Thirst	No bearing Slightly increases
6	Compromised Welfare	Pain, injury + Disease Discomfort Express normal behaviour	No bearing Slightly/strongly increases
7	Single Item	Fear + Distress	No bearing/mixed
